# Automated surveillance of 911 call data for detection of possible water contamination incidents

**DOI:** 10.1186/1476-072X-10-22

**Published:** 2011-03-30

**Authors:** Adam J Haas, Darcy Gibbons, Chrissy Dangel, Steve Allgeier

**Affiliations:** 1 CSC, 4701 Creek Road, Suite 250, Cincinnati, OH 45242, USA; 2 CSC, 6101 Stevenson Avenue, Alexandria, VA 22304, USA; 3 US Environmental Protection Agency, 26 W. Martin Luther King Dr., Cincinnati, OH 45268, USA

## Abstract

**Background:**

Drinking water contamination, with the capability to affect large populations, poses a significant risk to public health. In recent water contamination events, the impact of contamination on public health appeared in data streams monitoring health-seeking behavior. While public health surveillance has traditionally focused on the detection of pathogens, developing methods for detection of illness from fast-acting chemicals has not been an emphasis.

**Methods:**

An automated surveillance system was implemented for Cincinnati's drinking water contamination warning system to monitor health-related 911 calls in the city of Cincinnati. Incident codes indicative of possible water contamination were filtered from all 911 calls for analysis. The 911 surveillance system uses a space-time scan statistic to detect potential water contamination incidents. The frequency and characteristics of the 911 alarms over a 2.5 year period were studied.

**Results:**

During the evaluation, 85 alarms occurred, although most occurred prior to the implementation of an additional alerting constraint in May 2009. Data were available for analysis approximately 48 minutes after calls indicating alarms may be generated 1-2 hours after a rapid increase in call volume. Most alerts occurred in areas of high population density. The average alarm area was 9.22 square kilometers. The average number of cases in an alarm was nine calls.

**Conclusions:**

The 911 surveillance system provides timely notification of possible public health events, but did have limitations. While the alarms contained incident codes and location of the caller, additional information such as medical status was not available to assist validating the cause of the alarm. Furthermore, users indicated that a better understanding of 911 system functionality is necessary to understand how it would behave in an actual water contamination event.

## Background

Drinking water contamination incidents can pose a significant public health risk when they are not detected in time to enact measures to reduce exposures and mitigate the spread of contaminated water in a utility's distribution system [1,2]. Several documented cases of water contamination incidents have concluded that the monitoring of health-seeking actions pursued by the general public may have allowed for earlier detection of contaminated water. Automated surveillance and astute clinician disease reporting are techniques that can monitor the health-seeking behaviors. This article illustrates examples of automated surveillance for minimizing the consequences during a contamination incident.

The *Salmonella *outbreak in Alamosa County began with a case report to the Alamosa Public Health Nursing Service on March 6, 2008, leading to the initiation of a preliminary investigation. By March 14, the number of cases reported had increased to 19. The epidemiological investigation did not find a common food source for all cases, although several had eaten at a high volume restaurant where the index case had worked. The critical piece of information was that of the five infants infected, all had been fed with powdered formula mixed with tap water and had no other common exposure. On March 19, 2008, drinking water samples were tested for total coliform bacteria through the use of a quick screening test; the results were positive. Though this test was not confirmatory, city officials decided to issue a bottled-water order at this stage of the incident. Beginning with the index case and continuing through April 29, 436 cases from this outbreak were documented. Based on a multiplier used by the Centers for Disease Control and Prevention (CDC) which indicates that for every case that presents for care, another 30 cases are likely to have occurred, an estimated 12,000 people were affected during this incident [3]. Automated surveillance may have resulted in sampling commencing sooner than five days after the first documented cases and consequences may have been less severe for Alamosa.

Another instance where automated surveillance may have provided an early warning of a water contamination incident occurred during a *Cryptosporidium *outbreak in Milwaukee, WI. In this case, a nursing hotline began to receive a dramatic increase in calls for cases of diarrhea on April 2, 1993 and the local emergency department had a peak of patients with similar symptoms on April 4^th ^[4]. Five days elapsed before a boil water advisory was issued on April 7, 1993; over 400,000 people were affected [5]. A retrospective study of two waterborne outbreaks involving *Cryptosporidium, E. coli *O157:H7, and *Campylobacter *in Canada indicated syndromic surveillance of over-the-counter medication sales would have provided an early indicator of contamination [6]. Close monitoring of developing public health incidents through a partnership between health agencies and the water utility can serve to reduce the timeline for determining whether contaminated water is the source of an incident and could prevent additional exposures to the contaminated water [7].

To address the risk of contamination of drinking water systems, the U.S. Environmental Protection Agency (EPA) began conceptual design of a multi-component contamination warning system in 2004, which culminated in the development of the *WaterSentinel System Architecture *[8]. Cincinnati, Ohio was chosen as the first city to demonstrate this conceptual design and deployment of the Cincinnati contamination warning system was substantially complete in December 2007. The public health surveillance component was one of the primary monitoring and surveillance components implemented under the pilot program. A partnership was formed between the Greater Cincinnati Water Works and key representatives of several local public health agencies, including the Cincinnati Health Department, Hamilton County Public Health, the Cincinnati Fire Department, and the Drug and Poison Information Center. Personnel supporting the component were responsible for conducting investigations of public health surveillance alarms produced by automated surveillance systems, and for participating in drills and exercises which simulated drinking water contamination incidents. Additional details on the design of the Cincinnati contamination warning system can be found in *Water Security Initiative: Cincinnati Pilot Post-Implementation System Status *[9].

During a water contamination incident, public health officials may expect that health-seeking behavior of individuals exposed to contaminated water would largely depend on the timing of symptom onset, severity of symptoms, and nature of the symptoms. These factors are a function of the type of contaminant introduced into the water supply, and are anticipated to have a significant impact on how the public would react to an incident. Some of the expected symptoms following exposure to a contaminant may include respiratory or gastrointestinal distress, skin irritation, or neurological symptoms. The following list includes a variety of actions that may be pursued by individuals seeking medical treatment for systems developed during an incident:

• Call 911

• Call a medical help line (e.g., nursing hotline or Poison Control Center)

• Drive to an emergency department

• Schedule an appointment with a primary care physician

• Purchase over-the-counter medication

Automated surveillance systems which are designed to monitor any of these public health data streams can provide an early warning when an established baseline threshold has been exceeded, signaling a possible contamination incident. To provide robust coverage, a public health surveillance component designed to monitor a variety of public health data streams would be capable of detecting both acute and gradually developing events. Acute events result from fast-acting contaminants that generally have an onset of symptoms after exposure within ten minutes to three hours, while one to several days may elapse between exposure to a contaminant and the onset of symptoms for gradually developing events. The term fast-acting contaminant describes any possible water contaminant that produces rapid onset of symptoms, within minutes to hours following exposure to an acutely harmful dose. Since, for purposes of the contamination warning system, the syndromes monitored are the same for both acute and gradually developing events caused by biological, radiological, or chemical contaminants, the timeliness of anomaly detection coupled with subject matter expertise during subsequent alert investigations determines what type of contaminant(s) may be responsible.

**Figure 1:**
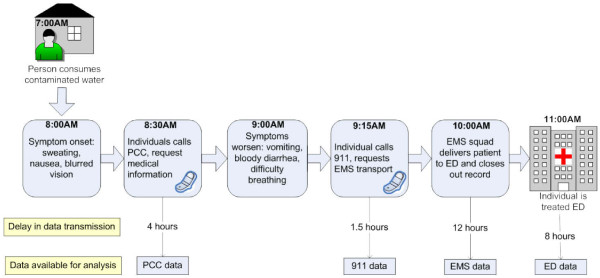
**Example Chemical Contamination Scenario (Carbamate Pesticide)**.

An example timeline for a contamination scenario is depicted in Figure 1, demonstrating symptom onset, actions of an exposed individual, and the unique data outputs which can be analyzed by public health surveillance systems. The timeline illustrated in this figure is based on the expected symptom onset and potential health-seeking actions that would occur following exposure to a carbamate pesticide. Note that Poison Control Center (PCC) data entry is initiated by poison control specialists (into the National Poison Data System [10]) immediately upon receipt of a call to the hotline. However, there is a four-hour latency period between case occurrence and case upload prior to when statistical analysis occurs to allow sufficient time for completion of data entry. Typically, follow-up phone calls occur after the initial hotline call is received to track the medical status of the caller. 911 data is entered by call dispatchers into the software system immediately upon receipt, and is available for analysis in approximately 1.5 hours following transmission to the surveillance system. Emergency Medical Services (EMS) data upload is delayed until an EMS unit closes out the record. Emergency department data upload is also dependant on the time that a patient's case record is completed and uploaded for analysis. The bottom portion of the figure depicts example time delays prior to when data is available for statistical analysis, based on the four data sources currently analyzed by automated surveillance systems within the public health surveillance component of the Cincinnati pilot. For PCC data, the delay in data transmission is the time delay for when data is available in NPDS for statistical analysis.

Prior to building the public health surveillance component of the Cincinnati pilot, a gap analysis was conducted to determine whether existing surveillance capabilities provided sufficient coverage for the spectrum of possible water contaminants. One automated surveillance tool, Real-time Outbreak and Disease Surveillance, was used by the local health departments to monitor emergency department visits. Real-time Outbreak and Disease Surveillance was designed to detect gradually worsening public health conditions, such as the onset of influenza season or a *Cryptosporidium *outbreak and was later replaced by EpiCenter. While emergency department data surveillance is programmed to execute on an hourly basis, alerts indicating anomalies in patient case load will only occur once per day (when anomalous conditions are present). Data entry specialists typically upload information from paper records of hospital cases from the previous 24 hours once per day; therefore, new electronic records often are not available for analysis more than once per 24 hour period. For this reason, EpiCenter was not optimal for detecting water contamination (either unintentional or intentional) with a fast-acting contaminant. For water contamination scenarios caused by fast-acting contaminants, community exposure would occur quickly, and symptoms would present rapidly; in most cases, less than 24 hours. Therefore, an automated surveillance system, capable of identifying possible contamination incidents due to fast-acting contaminants, (in a more timely fashion than EpiCenter could provide) was needed. One of the new systems implemented consisted of surveillance of 911 calls within the City of Cincinnati.

911 data offers several advantages over emergency department visit data which is commonly used by health departments for surveillance of possible public health outbreaks. Data is often more complete since the source provider consists of a few or even one dispatch center, rather than many hospitals. 911 data is available quickly, as the focus is to dispatch emergency services rather than to diagnose a patient. Additionally, geographic data provides the exact location of the illness, rather than the location of the hospital to which the patient was transported, which could service a large geographic area [11]. Besides improving spatial detection accuracy [12,13], precise geographic location information is critical to the response to suspected or confirmed water contamination. Water utility personnel involved in consequence management during response to an incident can use the location data of exposed individuals to identify the extent of the contaminated area and to trace the source of the contamination. This paper discusses the design, implementation, and application of automated, hourly 911 data surveillance for detection of possible water contamination in the Cincinnati contamination warning system.

## Methods

### Data Flow and Configuration

Cincinnati Police Department and Cincinnati Fire Department emergency dispatchers process 911 calls on a regular basis through Cincinnati's Motorola dispatch system. To improve standardization and electronic call tracking for 911 calls, a commercial software package, Priority Dispatch ProQA, was deployed in Cincinnati [14]. This software package assists 911 dispatchers in effective triage of calls by gathering a variety of health data in a systematic manner. The software directs dispatchers to assign incident codes to incoming calls according to the Medical Priority Dispatch System, which is a standard approved by the National Association of Emergency Dispatchers and utilized by over 3,400 dispatch centers in the US. Calls are triaged according to data elements, including chief complaint and geographic location (latitude/longitude) of the call. The caller initiates the complaint (i.e., breathing problems) and more specific information is attained by the dispatcher using prompts provided by the software program. In addition, instructions are provided to the caller until medical assistance arrives.

**Figure 2:**
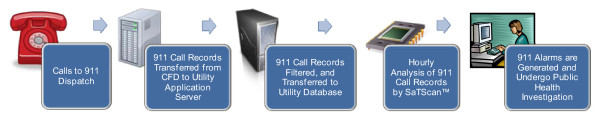
**911 Surveillence System Process**.

When call records are completed by dispatchers, the call data is stored in the Cincinnati Fire Department's dispatch system. Call detail data is then exported to a utility application server via Web services. Exported call detail information includes the call identifier, the incident type code, the date and time of the incident (call time and dispatch time), and the latitude and longitude of the incident location. All 911 calls are a part of the public record and not subject to Health Insurance Portability and Accountability Act privacy regulations. New call detail records are queried by the utility application server on a minute-by-minute basis. For call detail records that have incident type codes indicative of possible water contamination, as determined by epidemiologists from the Cincinnati Health Department and Hamilton County Public Health, and poison control specialists from the Drug and Poison Information Center prior to implementation of the system, a corresponding record is stored in a dedicated utility database for later reference by the 911 surveillance system. These incident types initially included symptoms pertaining to abdominal pain, allergies, breathing problems, cardiac or respiratory distress, headache, hemorrhaging, fainting, possible stroke, seizures, and unconsciousness; incident codes including symptoms for burns and blisters were added later following a call dispatch exercise conducted by Cincinnati Fire Department. Call detail records for these incident types remain on the server for only 28 days, after which they are removed. This timeframe allows for an adequate window to detect any alarms, which are documented for future analysis. This data flow process is depicted in Figure 2.

### Data Analysis with the Space-time Permutation Scan

The space-time permutation scan statistic was introduced by Kulldorff and colleagues for early detection of disease outbreaks without requiring knowledge of the population at risk [15]. The statistic relies entirely on case location and time data. Essentially, the method extends scan statistics to the temporal dimension. The method iteratively moves a cylindrical scan window across the study area, varying the cylinder size spatially and temporally. At each iteration, the Poisson generalized likelihood ratio that the cylinder contains an outbreak is evaluated. Over all iterations, the cylinder with the highest likelihood ratio constitutes the cylinder most likely to indicate outbreak. The highest likelihood ratio does not necessarily mean an outbreak has occurred. Statistical inference on the probability that the cylinder is the result of an outbreak relies on Monte Carlo hypothesis testing to account for multiple testing issues.

For the Cincinnati 911 surveillance system, the SaTScan™ software package was utilized to perform space-time permutation scans of filtered 911 calls in the City of Cincinnati. SaTScan™ is a free software package for spatial, temporal, and spatio-temporal analysis of data [16]. Prior to implementation of the 911 surveillance system, 911 data from the coverage area was analyzed to establish appropriate system parameters. Statistical analysis of historical data supported the use of a 21-day data set for the space-time permutation model. The space-time permutation model requires older data to evaluate the validity of the detected cluster. The more data involved in the analysis, the more confidence (i.e., lower p-value) there is in the existence of the cluster. At the same time, too much data may also result in lower confidence in the cluster's existence due to a recurrence of earlier dates experiencing clustering in the location [17].

To determine the most appropriate amount of historical data to include in the analysis, SaTScan™ compared data sets consisting of different lengths of historical data (four weeks, three weeks, two weeks, one week, six days, five days, two days). The dates chosen for the analysis were the three separate dates that had a cluster in the historical analysis and also generated an alert based on a parallel analysis of EMS data using the CDC's Early Aberration Reporting System [18]. Based upon the results of the three analyses (each of which compared the seven time periods of historical data described above), the most appropriate amount of historical data for space-time permutation was either three weeks or two weeks.

The three-week and two-week data sets both detected the targeted clusters with small p-values and high recurrence intervals. The recurrence interval indicates how often the detected cluster may occur by chance. If the recurrence interval is three months, then the expected number of false positives is one every three months. The three-week analyses had slightly lower p-values than the two-week analyses, and therefore was the recommended length of historical data to include in order to balance statistical significance with the recurrence interval.

For the public health surveillance component of the Cincinnati pilot, analysis of 911 data is executed hourly and the algorithm executes on a rolling three-week data set of 911 call detail records each analysis cycle. The analysis results provide the location and size of likely event clusters across the entire data set, sorted by the p-value. The p-value is evaluated over 999 Monte Carlo simulations.

### Alarm Notification

A 911 alarm will only be generated when the alert notification logic, as established for the public health surveillance component, is met. The initial design included alerting logic steps one through three below, to eliminate notifications from subsequent analyses that duplicated recent results. A fourth alerting logic step was later added to reduce alarms. The current alerting logic for identifying 911 alert conditions is:

1. If the space-time permutation scan identifies a candidate cluster with p-value < 0.0250 for a given day

2. If previous analyses have not already generated an alert for the exact cluster center identifier (911 call identifier closest to cluster center) for the given day

3. If the analysis does not measure the candidate cluster center point as being within the boundary of any previously alerted cluster(s) for a given day (distance from candidate alert-worthy cluster center to previously-alerted cluster center(s) is less than said previously-alerted cluster's radius)

4. If the event count (number of 911 calls) associated with the candidate cluster is > 16

When an analysis of 911 call details demonstrates that the data meet the alarm criteria, an email is transmitted by the 911 surveillance system to local public health epidemiologists/disease investigators. The email notification contains the cluster details such as the p-value, radius, centroid, number of cases in the cluster, and the number of cases expected in the scan window. Additionally, incident codes, HIPAA-compliant age, gender, and dispatch address (when available) for each case are provided for assessment of similarities by investigators. In the event of an alarm, a set procedure as outlined in the *Water Security Initiative: Interim Guidance on Developing an Operational Strategy for Contamination Warning Systems *is implemented for alarm review and verification of whether a water contamination incident is possible [19].

### Public Health Surveillance User Interface

Upon receiving the alarm email, the investigator will log onto a *Public Health Surveillance User Interface *to evaluate the cluster. The User Interface provides Web access to the analysis details for the both the 911 and EMS surveillance systems. The 911 analysis details provided in the alarm email are included on the home page, along with a history of 911 alarms. While location information is provided in 911 alarm notification emails, local public health partners requested the ability to visualize the data in a geographic information system (GIS) tool. Therefore, a modification was introduced which allows system users to view alarm details by clicking on a link within the User Interface which provides a GIS display. Currently, the bounding circle of the alarms in addition to locations and select information of the cases generating the alarms are exported in KML and displayed in Google Earth - a free GIS platform.

Upon receipt of a 911 alarm, the public health investigator will confirm proper coding of underlying data and check other public health surveillance systems for any corresponding trends. At this point, the investigator may be able to determine that the alarm is not a valid contamination event and close the investigation. If possible contamination is still suspected, the investigator will then contact the Water Utility Emergency Response Manager, and convene a conference call with other public health officials and the Drug and Poison Information Center. This team will then discuss the data associated with the alarm and the water utility will conduct an internal investigation and report to the public health team while following the utility's Consequence Management Plan.

## Results

Performance data for the 911 surveillance system was collected from January 2008 through June 2010. Data transmission times are provided in the analysis below, as well as alarm characteristics, such as frequency of alarms, number of cases in an alarm, distribution of alarms, and alarm area.

### Data Transmission

**Figure 3:**
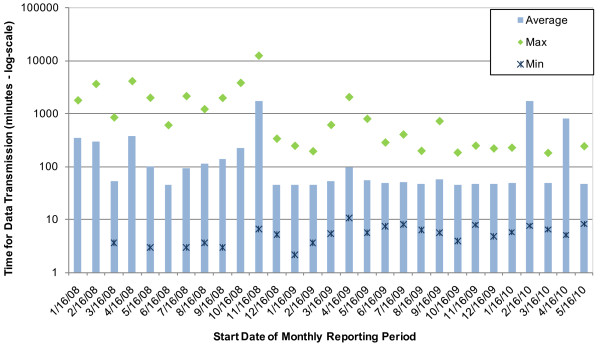
**911 Surveillance System - Time for Data Transmission**.

The time for data transmission, defined as the time that elapses between upload of 911 call records to the Cincinnati Fire Department server and subsequent transmission time to the utility application server for filtering, was calculated. As depicted in Figure 3, the time for data transmission of 911 call records ranged from 45 - 1706 minutes during the evaluation period, with an overall average transmission time of 225.6 minutes (~4 hours) and an overall median time of 47.6 minutes. The graph demonstrates that the data transmission time was typically between 45 and 100 minutes during most reporting periods. Given the median data transmission time, 911 alerts may be generated approximately 1 - 2 hours after call volumes had increased if individuals were exposed to contaminated water.

Occasional (seven) long delays (> 24 hours) in data transmission were caused by network outages, which caused downtime of the interface that transmits call records from the Cincinnati Fire Department server to the utility application server. Until this interface is manually restarted, data transmission cannot occur. Specifically, one notably long period of interface downtime (~9 days) occurred between November 25, 2008 and December 4, 2008, which was the result of network instability. During this time period, transmission of all records from the Cincinnati Fire Department server to the utility application server was impeded. This event noticeably increased the average transmission time for the November 16, 2008 reporting period.

During two reporting periods later in the evaluation timeline, longer data transmission times also occurred. In the February 16, 2010 reporting period, the 911 interface experienced a seven-day outage which delayed data transmission. Later, in the April 16, 2010 reporting period, the utility's 911 subscription expired, which caused a five-day delay in data transmissions between May 1, 2010, and May 6, 2010, when the issue was resolved.

### Frequency of Alarms (pre/post new alerting logic step implementation)

To date, there have been no true alarms generated by the 911 surveillance system. All alarms generated by the 911 surveillance system as during the evaluation period are considered false alarms, meaning that they did not indicate possible water contamination or other public health events.

**Figure 4:**
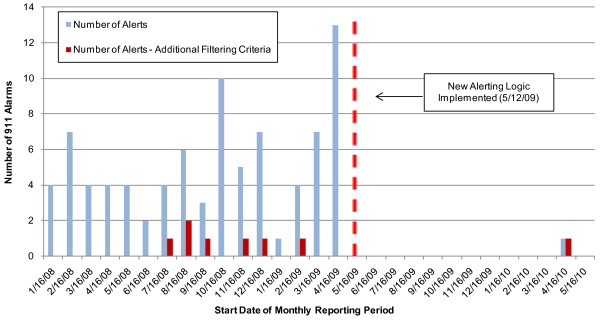
**911 Alarms per Reporting Period**.

Prior to the addition of the fourth alerting logic step, a total of 85 alarms were generated by the 911 surveillance system, or an average of five alarms per month. All alarms were determined to be the result of background variability and no apparent temporal trend in alarm frequency was observed (Figure 4).

Initially, the only limiting condition on alarm notifications was a maximum p-value of 0.025. This resulted in detection of many statistically significant anomalies that were of little concern to public health officials because there were so few cases in most alarms. The SaTScan™ configuration, combined with the first three alerting logic steps, resulted in an average of over five alarms per month between January 2008 and May 2009. This alarm rate was determined to be too frequent by the public health partners; therefore, the alarm history was examined to evaluate possible additional alerting logic that could be applied the 911 alarm notifications. Approximately 14 months of data was reviewed retrospectively to analyze factors such as p-value of the alert clusters, cluster radius per 911 alert, and number of 911 calls per alert. Since a significant number of the alarms contained very few cases (i.e., low number of 911 calls) and the public health partners were most interested in large events, the fourth alerting logic step was implemented in May 2009. Historical data analysis of alarms received indicated requiring a minimum of 17 calls for notification would have reduced the number of alarms received from 85 to 7 prior to implementation, which was acceptable to the public health partners.

**Figure 5:**
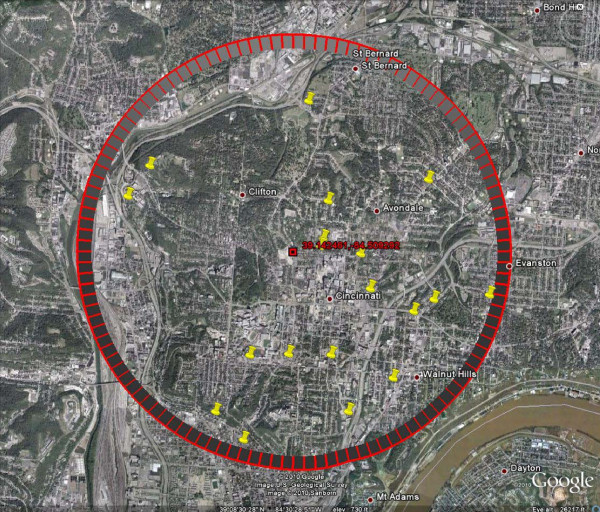
**Google Earth Display of April 2010 911 Alarm**.

Following implementation of this additional alerting criteria (post May 2009), only one 911 alarm occurred (see Figure 5). The alarm involved 19 calls over an area of 29 square kilometers. Notably, the lower alarm frequency post-May 2009 reduces the overall level of effort expended on alarm investigations and increases the likelihood that all alarms will be investigated (Allgeier S, Haas A, Pickard B: Optimizing Alarm Occurrence in the Cincinnati Contamination Warning System, submitted). If the new alerting threshold is applied to 911 data pre-May 2009, only seven alarms would have occurred. While the post-May 2009 alerting logic increases the acceptability of the surveillance system by greatly reducing the number of false alarms, it also lowers detection sensitivity and introduces the possibility that events eliciting fewer than 16 calls will be missed.

### Number of Calls in an Alarm

**Figure 6:**
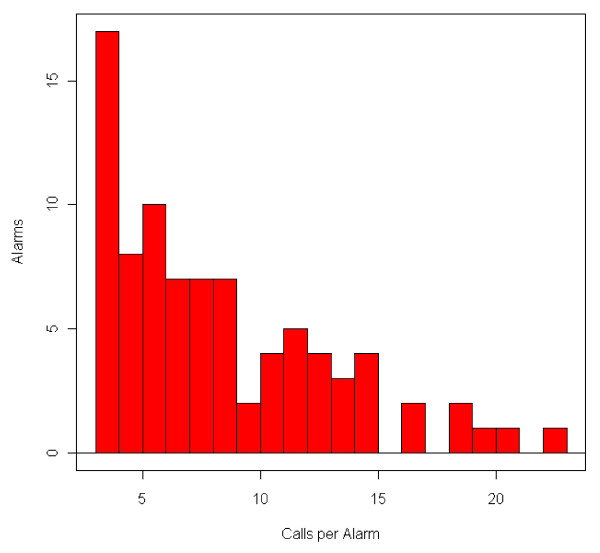
**Histogram of Number of Calls Per Alarm (n = 85)**.

The histogram presented in Figure 6 demonstrates the range in number of calls (i.e., cases) for alarms that occurred during the evaluation period. Over 90% of alarms contained 15 or fewer calls, and the central tendency of the number of calls per alarm was between 5 and 12 calls. The 911 surveillance system generated an alert on September 14, 2008 during the City of Cincinnati windstorm (a remnant of Hurricane Ike). This alert contained the highest number of 911 calls of any alert during the evaluation period - a total of 34 calls. This alert was excluded from the histogram for visualization purposes.

### Spatial Coverage of Alarms

Analysis of the geographic area covered by the 911 surveillance system was conducted to examine the distribution of 911 alerts within the City of Cincinnati. The analysis of empirical data contains a map of the surveillance area with an overlay of 911 alarms that occurred during the evaluation period.

**Figure 7:**
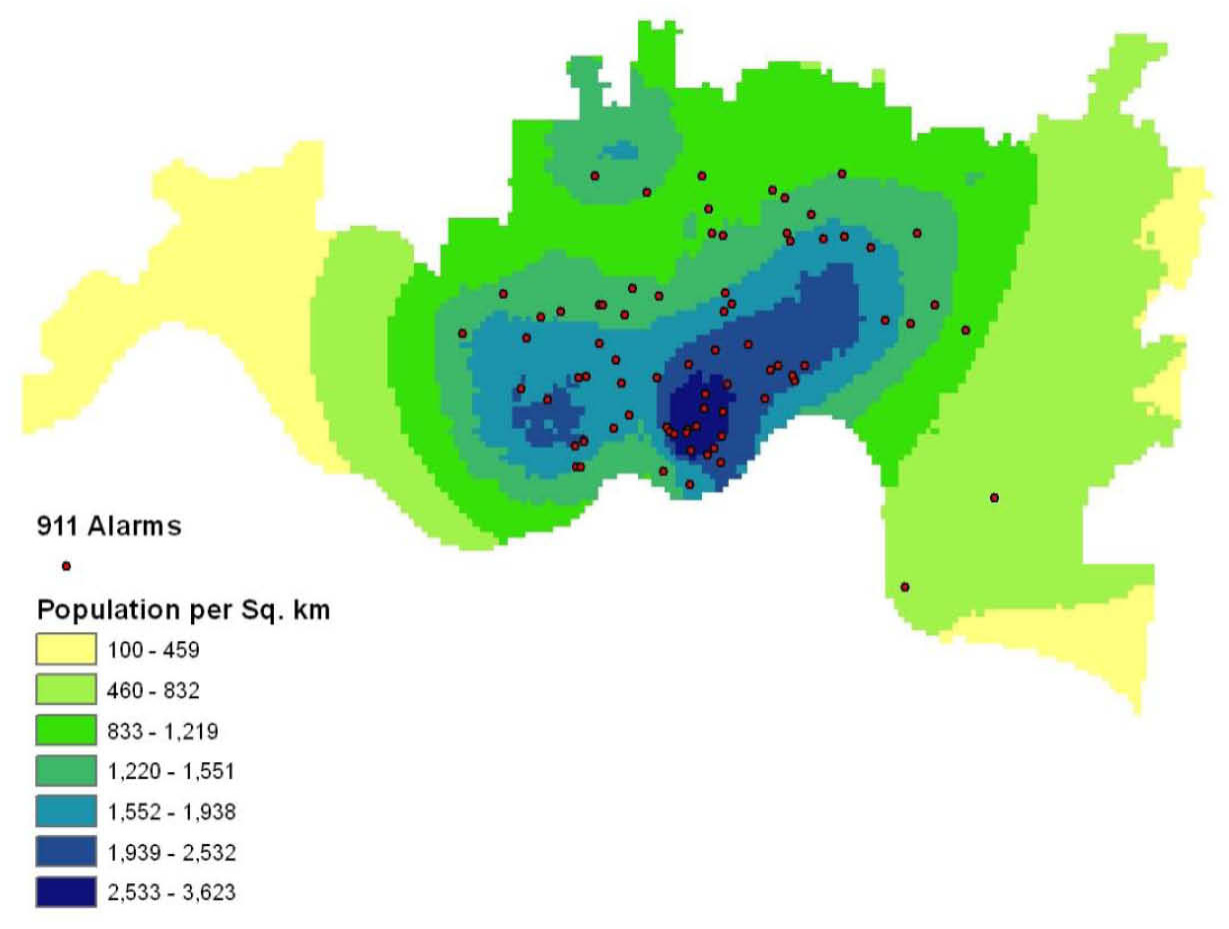
**Spatial Distribution of 911 Alarms in City of Cincinnati (n = 86)**.

During the evaluation period, a total of 86 alarms were generated. Figure 7 illustrates that these alarms were spatially distributed across the City of Cincinnati. Alarms were clearly more concentrated in areas of higher population density, as expected, since all alarms observed were false alarms and did not indicate a possible contamination or public health event. Most alarms were contained within the spatial area where the population density is greater than 1,219 individuals per square kilometer while no alarms occurred in areas with a population density less than 460 individuals per square kilometer.

### Spatial Extent of Alarms

Analysis of 911 alarms produced during the evaluation period demonstrates that most alarms were generated when a few calls co-occurred within close proximity in densely populated areas, or when many calls occurred over larger geographic areas where fewer calls were expected. Alarms with lower call density covered a larger alarm area, and those with higher call density covered a smaller alarm area.

Table 1 includes summary statistics related to the spatial extent of 911 alarms that occurred during the evaluation period. The alarm area represents a bounding circle of all calls contained in an alarm. Alarm area was calculated using the alarm radius, which is the distance from the alarm centroid (central point of all calls contained in the alarm) to the furthest call from the centroid.

**Table 1:** Statistical Analysis of Spatial Extent of 911 Alarms (n = 86)

	**Alarm Area (km**^**2**^**)**	Number of Calls	**Density (calls/km**^**2**^**)**
Average	9.22	9	28.54

Minimum	< 0.0003^1^	3	0.08

Maximum	253.21	34	995.22

^1 ^The minimum alarm area was less than SaTScan's™ minimum distance threshold (minimum radius = 0.01 km) which translates to a minimum alarm area of 0.0003 km^2^.

**Figure 8:**
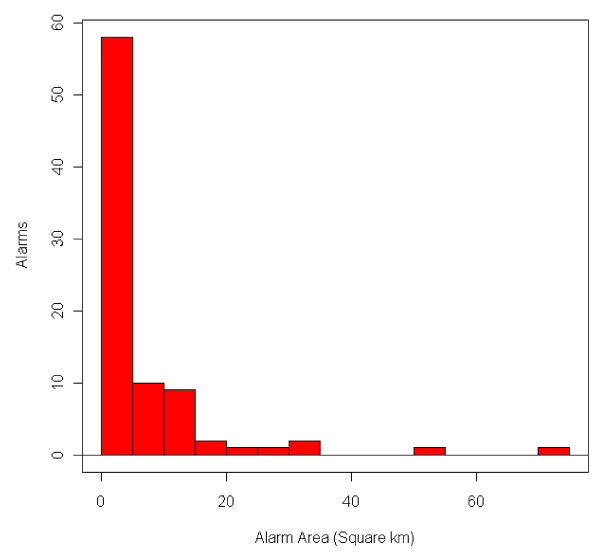
**Histogram of 911 Alarm Areas (n = 85)**.

The average 911 alarm area - 9.22 square kilometers - was small. The range in call density for 911 alarms was 0.08 - 995.22 calls per square kilometer. This range illustrates the upper and lower bounds of sensitivity of the 911 surveillance system based on the default alerting parameters. Tight call clustering is generally necessary for an alarm to be generated, which is supported by the fact that 80% of 911 alarms encompassed an area less than 10 square kilometers in size. Furthermore, 33% of 911 alarms covered an area less than 2 square kilometers. The histogram presented in Figure 8 demonstrates that the majority of 911 alarms covered small geographic areas. The 911 alert with the largest alarm area (253.21 square kilometers) also had the most calls in the alarm and was excluded from the histogram for visualization purposes. Of the other seven alarms which met the revised alert filtering criteria, the average alarm area was 36.36 square kilometers. Expectedly, since all of the alarms were false positives, alarms featuring a greater number of calls also covered a larger geographic area.

## Conclusions

In general, the public health partners indicated that the 911 surveillance system provided timely alerts and the use of a standardized software system by 911 call operators to enter 911 call data allowed for consistent coding. The speed of information (alarms containing location data) from 911 surveillance may be valuable for detection of fast-acting contaminants, but participants suggested that a better understanding of the functionality of the 911 surveillance system would be necessary to assess how the system may perform during an actual contamination incident. An increase in pre-implementation training was cited as a major factor that would have been beneficial to the initial process of investigating 911 alerts. One weakness identified by the system users was the limited amount of medical information available in the 911 alarm details. Though the incident code for each call is available, and location data demonstrates the degree of clustering among the cases, there is limited additional caller information (i.e., medical status) for investigators to consider when determining whether an alarm suggests possible water contamination. Despite this observation, additional surveillance systems utilized within the public health surveillance component may provide supplemental information that could further inform investigations of possible water contamination incidents. Furthermore, the location information included with the alarm is sufficient to guide the investigation.

During the evaluation, no water contamination was detected by this system or the other surveillance methods employed by the health departments which would directly assess how well the system met the design objective to detect fast-acting contaminants. However, participants in this monitoring program indicated even though an actual contamination event has not yet occurred which would test the ability of the surveillance system to detect an event, the speed of information from 911 surveillance is valuable for detection of fast-acting contaminants.

## Competing interests

The authors declare that they have no competing interests.

## Authors' contributions

AH and DG implemented data analysis and contributed to the interpretation of the results and writing of the manuscript. CD and SA authorized acquisition of data, participated in the study design and coordination, and helped draft the manuscript. All authors read and approved the final manuscript.
